# Simple synthesis of ^32^P-labelled inositol hexakisphosphates for study of phosphate transformations

**DOI:** 10.1007/s11104-017-3315-9

**Published:** 2017-06-27

**Authors:** Hayley Whitfield, Andrew M. Riley, Soulla Diogenous, Himali Y. Godage, Barry V. L. Potter, Charles A. Brearley

**Affiliations:** 1 School of Biological Sciences, University of Norwich, Norwich Research Park, Norwich, NR4 7TJ UK; 2 Medicinal Chemistry & Drug Discovery, Department of Pharmacology, University of Oxford, Mansfield Rd, Oxford, OX1 3QT UK; 3 Wolfson Laboratory of Medicinal Chemistry, Department of Pharmacy and Pharmacology, University of Bath, Bath, BA2 7AY UK

**Keywords:** Phytate, *neo*-, 1D-*chiro*-, *myo*-, *scyllo*-inositol hexakisphosphate, Phosphate

## Abstract

**Background and aims:**

In many soils inositol hexakisphosphate in its various forms is as abundant as inorganic phosphate. The organismal and geochemical processes that exchange phosphate between inositol hexakisphosphate and other pools of soil phosphate are poorly defined, as are the organisms and enzymes involved. We rationalized that simple enzymic synthesis of inositol hexakisphosphate labeled with ^32^P would greatly enable study of transformation of soil inositol phosphates when combined with robust HPLC separations of different inositol phosphates.

**Methods:**

We employed the enzyme inositol pentakisphosphate 2-kinase, IP5 2-K, to transfer phosphate from [γ-^32^P]ATP to axial hydroxyl(s) of *myo*-, *neo*- and 1D-*chiro*-inositol phosphate substrates.

**Results:**

^32^P-labeled inositol phosphates were separated by anion exchange HPLC with phosphate eluents. Additional HPLC methods were developed to allow facile separation of *myo*-, *neo*-, 1D-*chiro*- and *scyllo*-inositol hexakisphosphate on acid gradients.

**Conclusions:**

We developed enzymic approaches that allow the synthesis of labeled *myo*-inositol 1,[^32^P]2,3,4,5,6-hexakisphosphate; *neo*-inositol 1,[^32^P]2,3,4,[^32^P]5,6–hexakisphosphate and 1D-*chiro*-inositol [^32^P]1,2,3,4,5,[^32^P]6-hexakisphosphate. Additionally, we describe HPLC separations of all inositol hexakisphosphates yet identified in soils, using a collection of soil inositol phosphates described in the seminal historic studies of Cosgrove, Tate and coworkers. Our study will enable others to perform radiotracer experiments to analyze fluxes of phosphate to/from inositol hexakisphosphates in different soils.

**Electronic supplementary material:**

The online version of this article (doi:10.1007/s11104-017-3315-9) contains supplementary material, which is available to authorized users.

## Introduction

In consideration of the different forms of inositol hexakisphosphate identified in soils: 1D-*chiro*-, *myo*-, *neo*- and *scyllo*- ((Anderson 1964; Anderson and Malcolm 1974; Baker 1974, cited in Turner et al. 2002; Cosgrove 1962, 1963, 1966, 1969a, b; Cosgrove and Tate 1963; Halstead and Anderson 1970; L’Annunziata 1975; L’Annunziata and Fuller 1971; L’Annunziata et al. 1972; reviewed, Cosgrove 1980); Irving and Cosgrove 1982), it remains unclear what the biotic or abiotic origins of D-*chiro*-, *neo*- and *scyllo*-inositol phosphates are (L’Annunziata 2007; Turner and Richardson 2004; Turner et al. 2002). A limited number of studies have shown biotic contribution to the epimerization of unsubstituted inositols (Cosgrove 1969b; L’Annunziata 1975; L’Annunziata and Gonzalez 1977 (reviewed L'Annunziata 2007)), or have shown that chemical, and hence, geochemical, epimerization of *myo*-inositol pentakisphosphate is possible (Cosgrove 1972). It is clear from the foregoing that studies of soil phosphate transformations, particularly those arising from input of *myo*-inositol hexakisphosphate from plant sources, would be greatly enabled by the provision of ^32^P or ^33^P-labelled *myo*-inositol hexakisphosphate, and, indeed, of other inositol hexakisphosphates. Such materials would, with established extraction and separation techniques, allow facile determination of the exchange of phosphate between organic and inorganic pools and, with appropriate separation techniques, would allow study of transformations of different isomers of inositol hexakisphosphate. With labelled inositol hexakisphosphates and an increasing literature on the ‘pathways’ of *myo*-inositol hexakisphosphate degradation by phytases of different classes; cysteine phytase, histidine acid phytase, purple-acid phytase, β-propeller phytase (Konietzny and Greiner 2002), it would be possible to begin to describe ‘pathways’ of inositol hexakisphosphate turnover in soils and the contribution of different organisms to that turnover.

With these thoughts in mind, we have sought to synthesize ^32^P-labeled inositol hexakisphosphates by enzymic means. We have taken opportunity of a recombinant inositol pentakisphosphate 2-kinase (IP5 2-K, also known as IPK1) characterized (Banos-Sanz et al. 2012; Gonzalez et al. 2010; Gosein and Miller 2013; Sweetman et al. 2006). This enzyme transfers the gamma-phosphate) from ATP to the sole axial hydroxyl on carbon 2 of *myo*-inositol phosphates, and is believed to be the enzyme responsible for synthesis of *myo*-inositol hexakisphosphate in all kingdoms that make this molecule. We reasoned that the enzyme might be capable of transferring phosphate from ATP to the axial hydroxyl(s) of other inositols bearing equatorial phosphates.

**Fig. 1:**
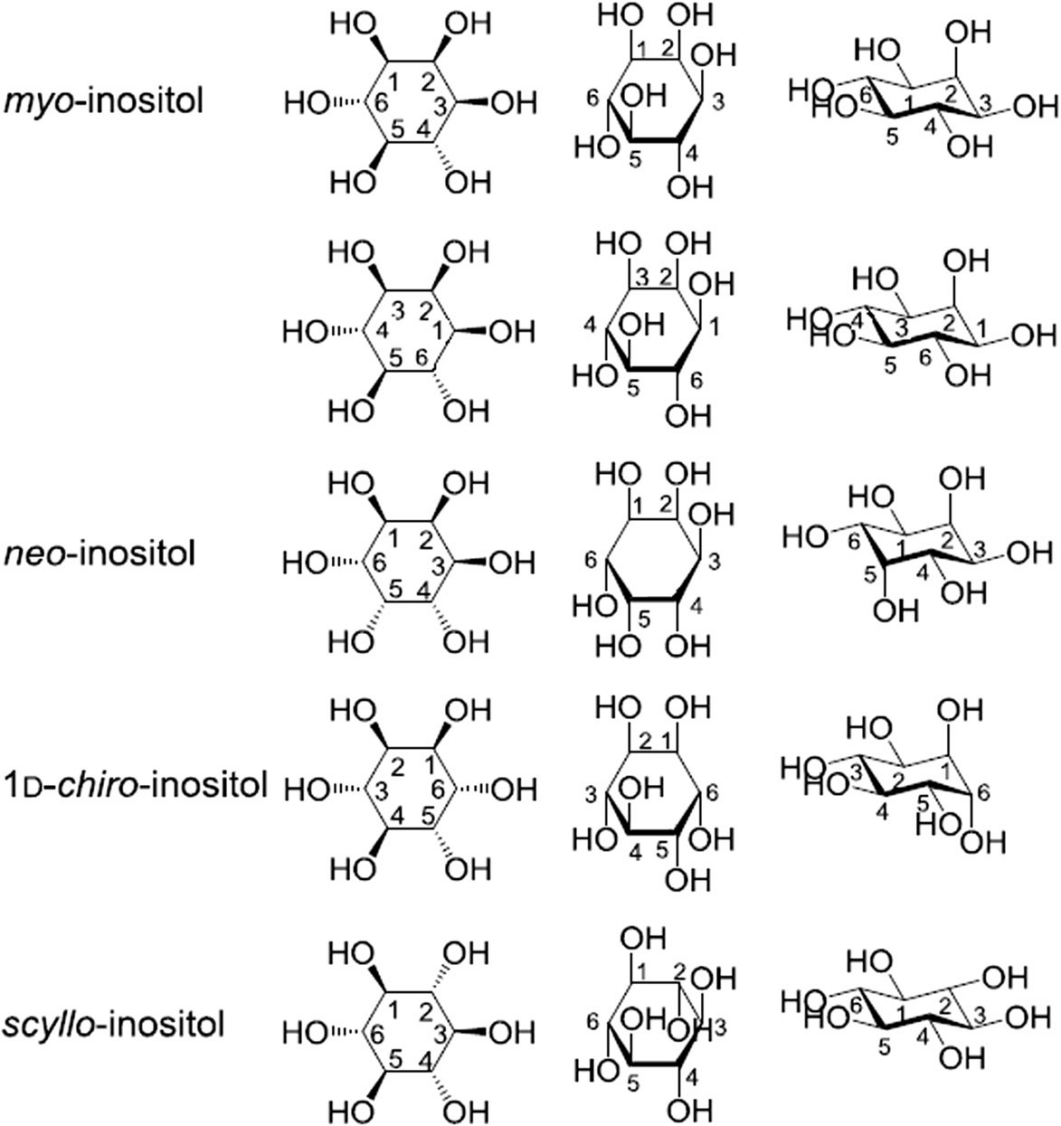
The structures of *myo*-, *neo*- *scyllo*- and 1D–*chiro*-inositols. The projections shown left to right are a Mills projection, a Haworth projection and a three-dimensional structure. Carbon atoms in the ring are numbered according to the IUPAC/IUPAC-IUB recommendations (1973, 1977). For *myo*-inositol, both 1L- (upper) and 1D- (lower) notation is shown

The structures of the different inositol ‘parents’ of the inositol phosphates used in this study are shown in Fig. 1. The rules for numbering of carbon atoms, and hence of inositol phosphates bearing phosphate substituents on particular carbons are given in the IUPAC-IUB rules (IUPAC-IUB 1973, 1977). For *myo*-inositol phosphates, only, a relaxation of the rules (NC-IUB 1989) allows numbering of carbons by the D- (1D) or L- (1L) nomenclature, e.g. to assist in delineating metabolic sequences. The 1D- and 1L- numbering of *myo*-inositol is shown in Fig. 1. Supplemental Fig. 1 shows symmetry aspects of the ‘parent’ inositols of the *myo*-, *neo*-, 1D-*chiro*- and *scyllo*-inositol phosphates discussed hereafter, while Supplemental Fig. 2 shows the structures of the substrates and products obtained therefrom.

For the assistance of researchers wishing to adopt these methods, we provide (Table 1) a summary of the methodologies used with an indication of their applicability and a reference to prior use of the method. We also list the figures in this manuscript in which the method is applied.

**Table 1:** Summary of methods of this study and their application to the study of inositol phosphate metabolism

Subject	Method	Comment	Description/Use	Reference
Stereochemistry of inositol phosphates		A comprehensive review of inositol (phosphate) chemistry and stereochemistry	Fig. 1	Thomas et al. 2016
Source of inositol phosphates	Complete synthesis or extracted from soils	The soil extracted inositol phosphates were the kind gift of Alan Richardson (CSIRO) from the personal stocks of the late Dennis Cosgrove, or were provided by Max Tate (The University of Adelaide)	Figs. 1, 2, 3, 4, 5, and 6; Suppl. Fig. 1	Diogenous 1999; Liu et al. 1999, 2001; Riley et al. 2006; Turner et al. 2012; Godage et al. 2013; collected works of Cosgrove, Tate and others (reviewed this manuscript)
Source of synthetic enzyme			Figs. 2, 3, 4, 5, and 6; Suppl. Fig. 1	Sweetman et al. 2006
Non-radioactive verification of enzyme specificity	Reverse-phase ion-pair chromatography with UV detection of nucleotides	Used to titrate enzyme concentration before use of radiolabel	Fig. 2	Caddick et al. 2008
Production of ^32^P–labeled inositol phosphates	Anion-exchange chromatography on Partisphere SAX columns with on-line detection by Cerenkov counting	Used to separate/verify reaction products	Fig. 3	Brearley and Hanke 1996a, b; Nagy et al. 2009; Stephens 1990
Substrate specificity/product profiles of different phytases	Anion-exchange chromatography on Partisphere SAX columns with on-line detection by Cerenkov counting	Can be used to separate/purify particular isomers	Fig. 4	Stentz et al. 2014
Non radioactive detection of inositol phosphates on acid gradients with UV detection	Anion exchange chromatography on CarboPac PA200 columns with post-column addition of ferric nitrate	A particularly robust separation method for higher inositol phosphates using volatile (HCl) or non-volatile (methanesulfonic) acid eluents. Could be combined with Cerenkov counting for radiolabeled inositol phosphates	Fig. 5	Phillippy and Bland 1988; Blaabjerg et al. 2010
Non radioactive detection of pentakisphosphates that can be generated from soil inositol hexakisphosphates	Anion exchange chromatography on CarboPac PA200 columns with post-column addition of ferric nitrate	A particularly robust separation method for higher inositol phosphates using volatile (HCl) or non-volatile (methanesulfonic) acid eluents. Could be combined with Cerenkov counting for radiolabeled inositol phosphates	Fig. 6	This manuscript
The structures of substrates and products of *At*IP5 2 K			Suppl. Fig. 1	This manuscript

## Methods

### Inositol phosphate substrates

The synthesis of *neo*-inositol 1,3,4,6-tetrakisphosphate (*neo*-Ins(1,3,4,6)P_4_), 1D-*chiro*-inositol 1,3,4,6-tetrakisphosphate (1D-*chiro*-Ins(1,3,4,6)P_4_), 1D-*chiro*-inositol 2,3,4,5-tetrakisphosphate (1D-*chiro*-Ins(2,3,4,5)P_4_), *myo*-inositol 1,3,4,5,6-pentakisphosphate (*myo*-Ins(1,3,4,5,6)P_5_), *scyllo*-inositol pentakisphosphate and of *neo*- and 1D-*chiro* inositol hexakisphosphates was described (Diogenous 1999; Liu et al. 1999; Liu et al. 2001; Godage et al. 2013; Riley et al. 2006; Turner et al. 2012)). *Myo*-inositol hexakisphosphate was obtained from Merck Millipore (Product No. 407125).

### Radioisotopes

ATP, [γ-^32^P]- 3000 Ci mmol^−1^ was obtained from PerkinElmer.

### Cloning and expression of AtIPK1

*Arabidopsis thaliana IPK1* (AGI number): At5G42810, was cloned from *Arabidopsis thaliana* Col-0 cDNA with forward primer *AAGTTCGTTTTCAGGGCCCG*ATGGAGATGATTTTGGAGGAGAA and reverse primer *ATGGTCTAGAAAGCTTTA*GCTGTGGGAAGGTTTTG (vector specific sequence in italics) using Phusion High Fidelity Polymerase (Thermo Scientific). Purified product (Wizard SV Gel and PCR Cleanup System, Promega) was inserted into pOPINF linearized with HindIII and KpnI (Berrow et al. 2007) by ligation independent cloning using In Fusion HD enzyme kit (Clontech). The vector adds a *N*-terminal hexahistidine tag to the recombinant protein. Recombinant plasmid was transformed into *E coli* Rosetta (DE3) (Novagen) and protein production induced from an overnight culture grown in LB containing 0.5% (*w*/*v*) glucose and ampicillin by transfer to LB containing 0.5 mM IPTG and ampicillin with further growth for 7 h at 25 °C.

### Protein purification

*At*IPK1 was purified according to (Banos-Sanz et al. 2012).

### Enzyme assays

For assays without radiolabel, inositol phosphate-dependent conversion of ATP to ADP was followed by reverse-phase ion pair HPLC and subsequent detection of nucleotides at 260 nm (Caddick et al. 2008). Briefly, 1 μg enzyme was incubated with 500 μM inositol phosphate and 50 μM ATP in 20 mM HEPES, pH 7.3, 1 mM MgCl_2_ for 2 h at 25 °C, the volume of the assay was 20 μL. Reactions were terminated by the addition of 1 μL of conc. HCl, followed after 5 min on ice by the addition of 50 μL water. Aliquots (50 μL) were analysed by HPLC.

For assays with radiolabel, ATP-dependent conversion of inositol phosphates to higher (more phosphorylated) species was followed by anion-exchange HPLC with on-line detection of ^32^P. Briefly, 1 μg enzyme was incubated with 500 μM inositol phosphate and 5 μM ATP in the presence of 0.37 MBq [γ-^32^P]ATP in 20 mM HEPES, pH 7.3, 1 mM MgCl_2_ for 1 h at 25 °C, the volume of the assay was 20 μL. The reaction products were diluted with water and approximately 1–5% of the products were analysed by HPLC.

### HPLC separation of inositol phosphates

Radiolabeled inositol phosphates were separated by anion exchange HPLC on Partisphere SAX columns eluted at a flow rate of 1 mL min^−1^ with a gradient derived by mixing solvent from reservoirs containing (A) water and (B) 1.25 M (NH_4_)_2_HPO_4_, adjusted to pH 3.8 with H_3_PO_4_, according to the following schedule: time (min), % B; 0, 0; 5, 0; 65, 100. Radioactivity was detected by Cerenkov counting in a Radiomatic A500 Series Flo Detector (Canberra Packard, Pangbourne, Bucks, UK) fitted with a 0.5 ml flow cell using an integration interval of 12 s (Hanke et al. 2012).

Non-labelled inositol phosphates were resolved by anion exchange HPLC on a 250 × 3 mm i.d. CarboPac PA200 column (Dionex UK, Ltd) and guard column 50 × 3 mm of the same material, eluted at a flow rate of 0.4 mL min^−1^ with gradients of either HCl or methanesulfonic acid (Blaabjerg et al. 2010). Inositol phosphates were detected after post-column addition of 0.1% (*w*/*v*) ferric nitrate in 2% HClO_4_ (Phillippy and Bland 1988) delivered at a flow rate of 0.2 mL min^−1^. The gradient for both eluents was (A) water, (B) 0.6 M acid: time (min), % B; 0, 0; 25, 100; 38, 100.

### HPLC separation of nucleotides

Aliquots of the products of enzyme assays were analysed according to (Caddick et al. 2008).

### Nomenclature

For the purpose of this article, the term ‘Ins’ with prefix 1D-*chiro*-, *myo*-, *neo*- or *scyllo*- is used as an abbreviation of the described inositol phosphate. Hence, *neo*-inositol 1,3,4,6-tetrakisphosphate is abbreviated *neo*-Ins(1,3,4,6)*P*_4_. It should be noted, however, that the numbering of phosphate substituents (of the carbon atoms to which they are attached) is not necessarily the same for different stereoisomers of inositol. The reader is referred to Shears and Turner (2007) for a concise description of terminology and to (Thomas et al. 2016) for a comprehensive review of inositol and inositol phosphate nomenclature and terminology.

## Results

*At*IP5 2-K can be used to synthesize a range of inositol phosphate epimers, verifiable by non-radioactive assay of inositol phosphate production

We have previously described the use of *At*IP5 2-K to synthesize *myo*-Ins(1,[^32^P]2,3,4,5,6)*P*_6_ from [γ-^32^P]ATP and *myo*-Ins(1,3,4,5,6)*P*_5_ (Nagy et al. 2009). Here, we have further examined the ability of *At*IP5-2 K to phosphorylate *myo*-Ins(1,3,4,6)*P*_4_. We did so, not only because *myo*-Ins(1,3,4,6)*P*_4_ possesses an axial 2-OH, but also because of the availability of the *neo*-inositol epimer, *neo*-Ins(1,3,4,6)*P*_4_ (Diogenous 1999). This inositol phosphate shares the plane of symmetry that bisects the *myo*-Ins(1,3,4,6)*P*_4_ molecule between C2 and C5, but also possesses a second axial hydroxyl on C5 which creates a *C*_2_-axis of rotational symmetry that bisects the C1-C6 bond and the C3-C4 bond (Supplementary Figure 1). The consequence of this is that single phosphorylation of C2 generates the same product as phosphorylation of C5, while in contrast phosphorylation of C2 of *myo*-Ins(1,3,4,6)*P*_4_ is not equivalent to phosphorylation of C5.

**Fig. 2:**
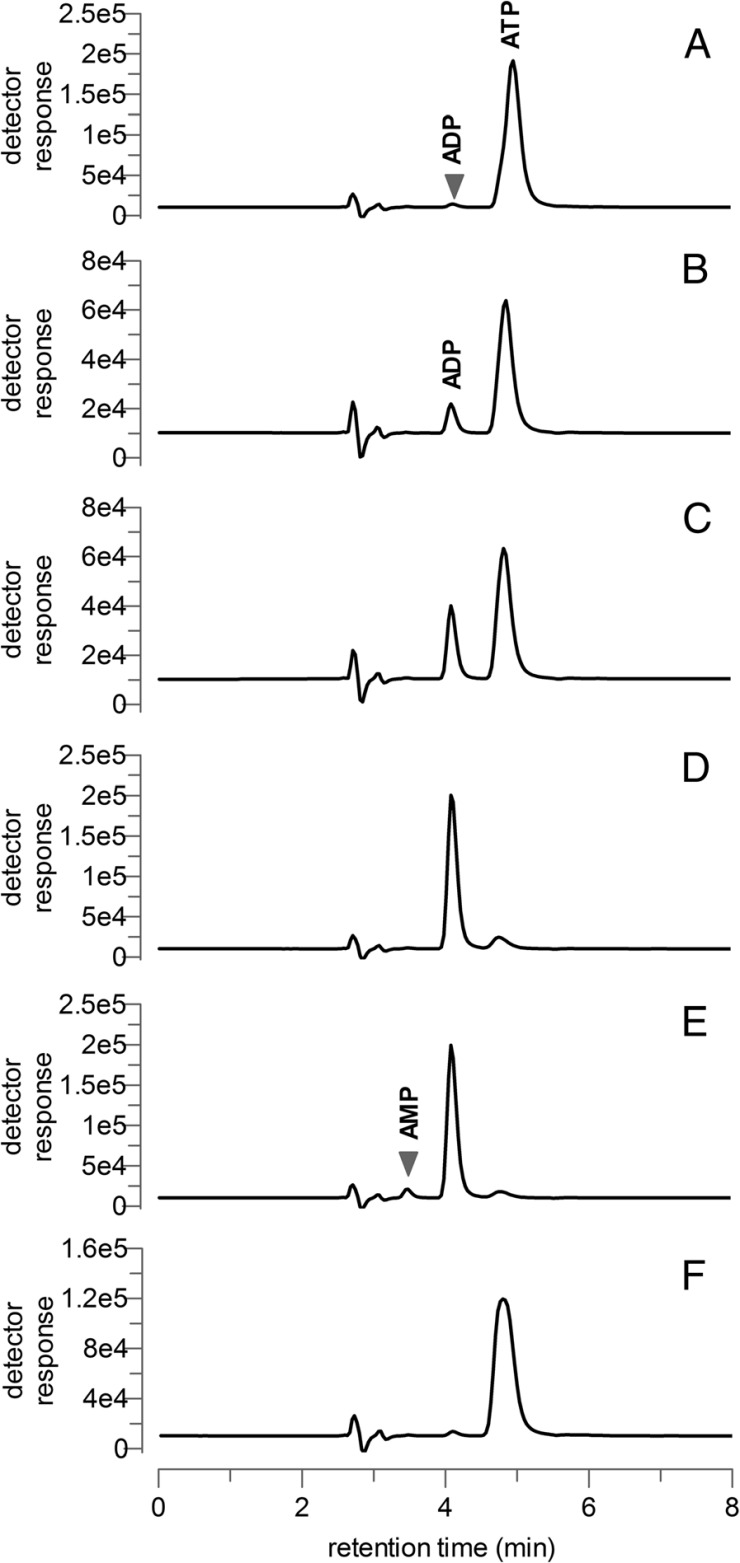
Phosphotransferase reactions catalysed by AtIP5 2-K. The nucleotide products of reactions of enzyme, ATP and different inositol phosphates were separated by ion-pair RP-HPLC and detected by absorbance at 260 nm. **a** no inositol phosphate; **b** *myo*-Ins(1,3,4,6)*P*_4_; **c** *myo*-Ins(1,3,4,5,6)*P*_5_; **d** *neo*-Ins(1,3,4,6)*P*_4_; **e** 1D–*chiro*-Ins(2,3,4,5)*P*_4_; **f** 1D–*chiro*-Ins(1,3,4,6)*P*_4_

We incubated *At*IP5 2-K with 500 μM inositol phosphate and 50 μM ATP. The products were resolved by reverse-phase ion-pair HPLC with detection of nucleotides at 260 nm (Fig. 2). Peak areas were integrated and the % of the nucleotide converted to ADP was calculated. A control incubation without inositol phosphate confirmed that *At*IP5 2-K is not a phosphatase; the 1.3% of nucleotide recovered as ADP is typical of the level of contamination of commercial ATP with ADP (Fig. 2a). Inclusion of *myo*-Ins(1,3,4,6)*P*_4_ increased ADP production, 12.1% of total nucleotide was recovered as ADP, without production of AMP (Fig. 2b), whereas for the physiological substrate *myo*-Ins(1,3,4,5,6)*P*_5_, included at 50 μM, 26% of nucleotide was recovered as ADP (Fig. 2c). Clearly, *myo*-Ins(1,3,4,6)P_4_ is a substrate, albeit a poorer one than *myo*-Ins(1,3,4,5,6)*P*_5_. In contrast, *neo*-Ins(1,3,4,6)*P*_4_ was a strong substrate with ADP production at 90.8% (Fig. 2d).

Similarly, inclusion of 1D-*chiro*-Ins(2,3,4,5)*P*_4_ at 500 μM resulted in 92% conversion of ATP to ADP (Fig. 2e), while 1D-*chiro*-Ins(1,3,4,6)*P*_4_ whose two hydroxyls, on C2 and C5 are equatorial (Supplementary Figure 1) was not a substrate for *At*IP5 2-K, with ADP representing 1.5% of the total nucleotide (Fig. 2f).

These results confirm the utility of *neo*-Ins(1,3,4,6)*P*_4_ and 1D-*chiro*-Ins(2,3,4,5)*P*_4_ as substrates from which higher *neo*- and 1D-*chiro*-inositol phosphates can be synthesized with *At*IP5 2-K. They further confirm the exclusive phosphorylation of axial hydroxyls by this enzyme (Gonzalez et al. 2010; Sweetman et al. 2006).

We additionally tested racemic mixtures of 1D/L-*neo*-Ins(1,2,4)*P*_3_ and 1D/L -*neo*-Ins(1,3,4)*P*_3_ as substrates in extended (16 h) incubations at 500 μM concentration with 50 μM ATP. We did not observe production of ADP, thus these molecules are not substrates despite possessing one and two axial hydroxyls at C5, and C2 and C5 respectively (data not shown).

### *At*IP5 2-K can be used to synthesize a range of ^32^P-labeled inositol phosphates

**Fig. 3:**
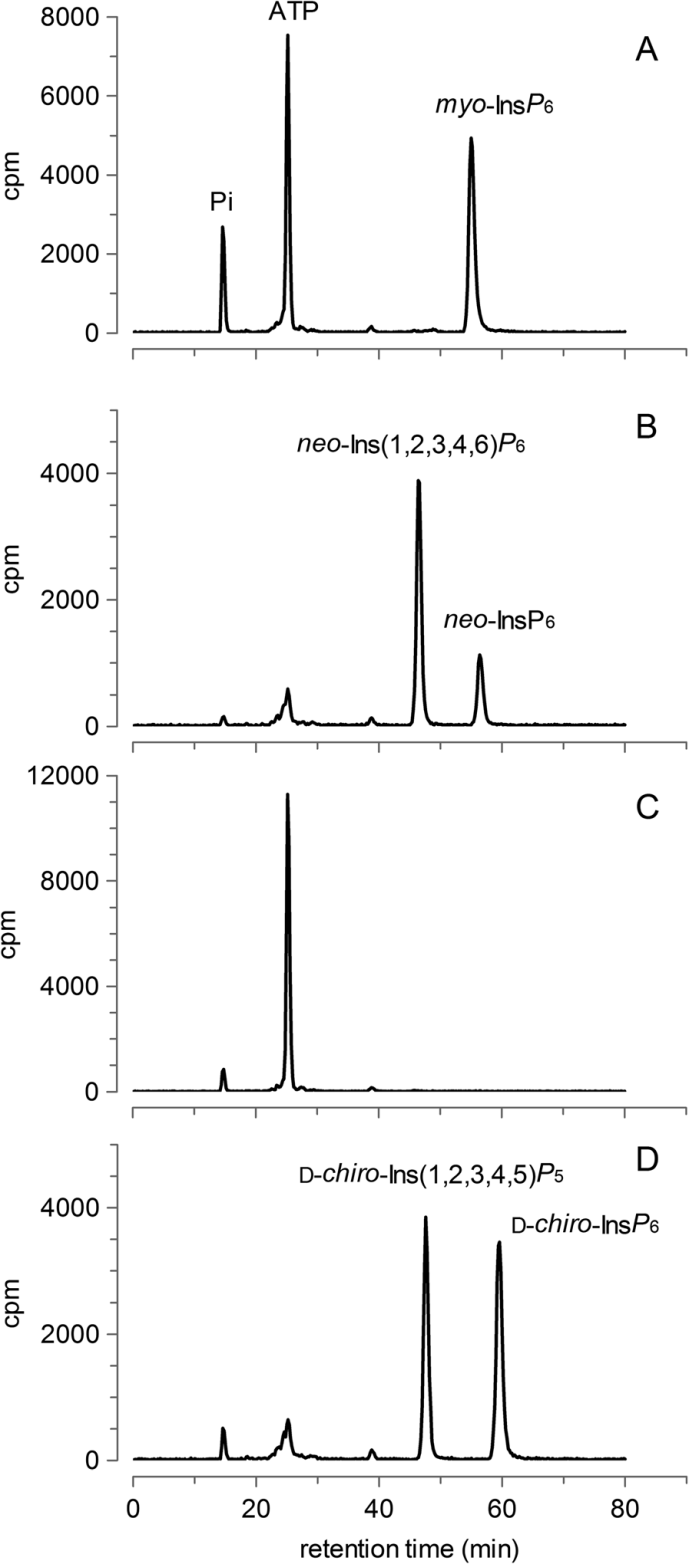
Synthesis of ^32^P-labelled inositol phosphates with AtIP5 2-K. The inositol phosphate products of reactions of enzyme, [^32^P]ATP and different inositol phosphates were separated by Partisphere Strong Anion Exchange HPLC and radioactivity estimated by on-line Cerenkov counting. **a** *myo*-Ins(1,3,4,5,6)*P*_5_; **b** *neo*-Ins(1,3,4,6)*P*_4_; **c** 1D–*chiro*-Ins(1,3,4,6)*P*_4_, **d** 1D–*chiro*-Ins(2,3,4,5)*P*_4_. The peaks labelled are the ^32^P labelled products or reactants (ATP)

Having established that *neo*-Ins(1,3,4,6)*P*_4_ and 1D-*chiro*-Ins(2,3,4,5)*P*_4_ are novel substrates of *At*IP5 2 K, we performed enzyme assays to produce ^32^P-labelled inositol phosphates. The substrate concentrations used were 500 μM inositol phosphate and 5 μM ATP. Reactions were terminated and the products spiked with additional ATP to allow online tandem UV-radioactivity monitoring of the chromatography (Fig. 3). We included *myo*-Ins(1,3,4,5,6)*P*_5_ as the canonical substrate and observed (Fig. 3a) in addition to a major peak of *myo*-Ins*P*_6_ eluting at a retention time of 55 min, a small peak of unidentified material eluting at 38.8 min, a major peak of unreacted ATP at 25.2 min and a peak of inorganic phosphate at 14.6 min. We note that others have reported impurities in commercial [^32^P]ATP that elute on Partisphere SAX columns with similar chromatographic mobility to *myo*-Ins*P*_4_s (Stephens 1990).

Provision of *neo*-Ins(1,3,4,6)*P*_4_ as substrate yielded (Fig. 3b) a major peak of label with the chromatographic property of Ins*P*_5_ at 46.4 min and a more polar (highly charged peak) with the chromatographic property of an Ins*P*_6_. This peak eluted at 56.4 min, slightly after *myo*-Ins*P*_6_. The trace also showed peaks of assumed impurity, ATP and inorganic phosphate. Considering the structure of *neo*-Ins(1,3,4,6)*P*_4_ and its *C*_2_ axis of rotational symmetry (Supplemental Figs. 1, 2), the two axial hydroxyls are superposable so there is only one possible Ins*P*_5_ product. IUPAC conventions recommend the naming of substituents by the lowest numbered locants, hence single phosphorylation of one axial hydroxyl of *neo*-Ins(1,3,4,6)*P*_4_ yields *neo*-Ins(1,2,3,4,6)*P*_5_ = *neo*-Ins(1,3,4,5,6)*P*_5_ with preferred use of the former name. The addition of ^32^P to an unlabeled substrate therefore yields *neo*-Ins(1,[^32^P]2,3,4,6)*P*_5_. The elution of a second more polar peak, we assume to represent the double phosphorylation of *neo*-Ins(1,3,4,6)*P*_4_ to give *neo*-Ins(1,[^32^P]2,3,4,[^32^P]5,6)*P*_6_.

We also tested 1D-*chiro*-Ins(1,3,4,6)*P*_4_ and 1D-*chiro*-Ins(2,3,4,5)*P*_4_ as co-substrates with [^32^P]ATP (Fig. 3c, d). Significantly, the former, which bears equatorial hydroxyls on C2 and C5, and lacks axial hydroxyls (Fig. 1; Supplemental Figs. 1, 2), was not a substrate and yielded peaks of unreacted ATP, inorganic phosphate and the assumed contaminant with retention time 39 min (Fig. 3c). In contrast, 1D-*chiro*-Ins(2,3,4,5)*P*_4_, like *neo*-Ins(1,3,4,6)*P*_4_, yielded ^32^P-labeled peaks with the chromatographic mobility of Ins*P*_5_, retention time 48 min; and Ins*P*_6_, retention time 59.5 min (Fig. 3d). Comparison of the retention time of peaks of Ins*P*_5_ and Ins*P*_6_ products (Fig. 3a–d) reveals that the Ins*P*_5_ and Ins*P*_6_ products of different epimers of inositol (tetrakisphosphate) are chromatographically distinct. Again, 1D-*chiro*-Ins(2,3,4,5)*P*_4_, another substrate with two axial hydroxyls, yielded products which, relative to the substrate, were singly and doubly phosphorylated by AtIP5 2-K. Moreover, 1D-*chiro*-Ins(2,3,4,5)*P*_4_ possesses a *C*_2_-axis of symmetry, here bisecting the C1-C6 and C3-C4 bonds, which superposes C1 and C2 substituents. Consequently, a common Ins*P*_5_ product is generated from phosphorylation of either axial hydroxyl and the product is 1D-*chiro*-Ins(1,2,3,4,5)*P*_5_, here 1D-*chiro*-Ins([^32^P]1,2,3,4,5)*P*_5_. The Ins*P*_6_ product is 1D-*chiro*-Ins([^32^P]1,2,3,4,5,[^32^P]6)*P*_6_.

### Different phytases yield characteristic product profiles from ^32^P-labeled *myo*-inositol hexakisphosphate

**Fig. 4:**
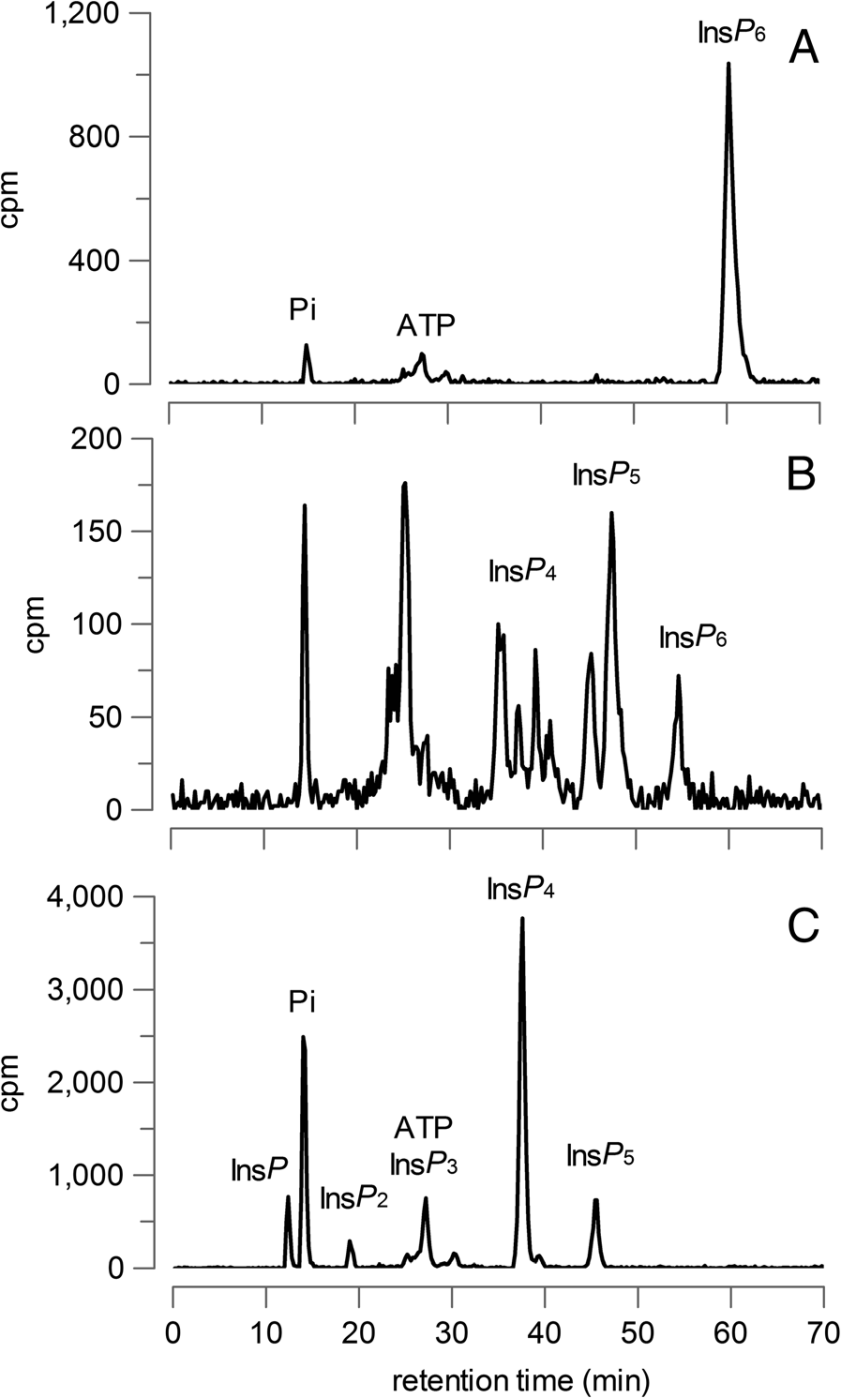
Separations of ^32^P labelled inositol phosphate products of dephosphorylation of *myo*-Ins(1,[^32^P]2,3,4,5,6)*P*_5_ by HAE (*Bacteroides thetaiotomicron* Minpp) and HD (*Aspergillus niger*) phytases. The products of reactions of **a** no enzyme control; **b** *Bt*Minpp and **c** *A ficuum* phytase were separated by Partisphere Strong Anion Exchange HPLC and radioactivity estimated by on-line Cerenkov counting. The positions of elution of representative classes of *myo*-inositol phosphate products and of contaminating ATP in the *myo*-[^32^P]Ins*P*_6_ preparation are indicated. The difference in retention time of inositol hexakisphosphate between different panels reflects the use of different Partisphere SAX columns for the separations

By way of illustration of how different phytases yield different product profiles from the same substrate, we show (Fig. 4) the products of progressive dephosphorylation of *myo*-Ins(1,[^32^P]2,3,4,5,6)*P*_6_ by histidine acid phytases of fungal and bacterial origin. We chose *Aspergillus ficuum* phytase, a D-3 phytase (data of Fig. 4c), where D-3 signifies the position of attack on *myo*-Ins*P*_6_ and HD is one of the canonical motifs, D (aspartate) being a proton donor that activates a water molecule that is responsible for the cleavage of scissile phosphate. The other phytase (data of Fig. 4b) is another histidine acid phytase, but is the archetype of a recently described HAE subclass where E (glutamate) is the likely proton donor. This enzyme generates three resolvable Ins*P*_5_ products from *myo*-Ins*P*_6_ (Stentz et al. 2014). These data reveal the utility of inositol hexakisphosphate labeled on the axial 2-position for study of phytases in vitro. Clearly, they indicate the potential utility of such compounds and such chromatography for study of soil processes.

### Separation of inositol hexakisphosphates identified in soils

**Fig. 5:**
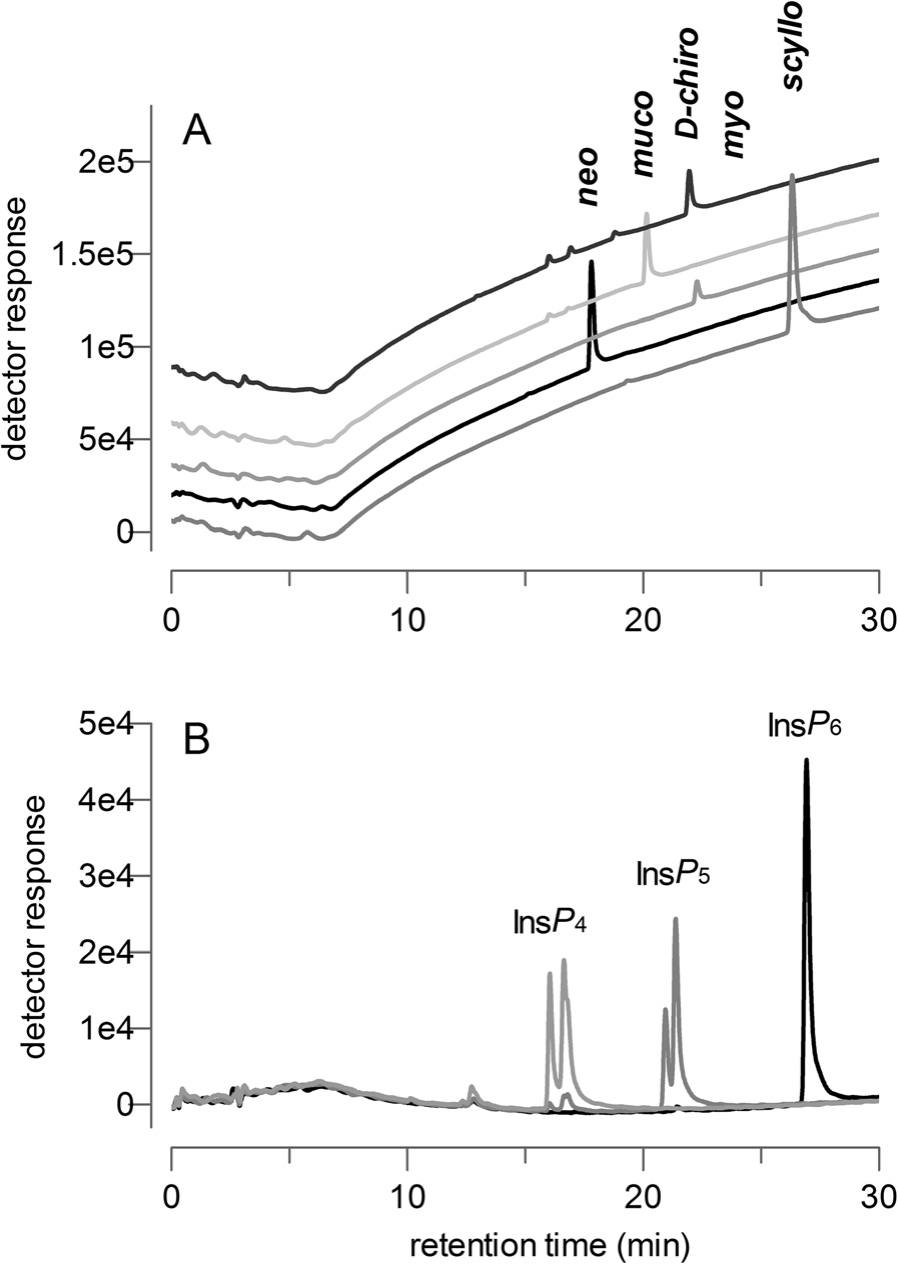
Separations of soil-representative inositol phosphates on a Dionex CarboPac PA200 column. The column was eluted with a gradient of HCl (**a**) or methanesulfonic acid (**b**) and inositol phosphates detected by post-column complexation with ferric nitrate in perchloric acid and subsequent detection at 290 nm. **a**, **b** Inositol phosphates were obtained from the laboratory of the late DJ Cosgrove. **a** the traces from individual injections (approximately 20 nmol) of different inositol hexakisphosphates are offset on the Y-scale to aid visualisation. **b** Samples of individual injections of synthetic *neo*-Ins*P*_4_ with retention time 16–17 min, *neo*-Ins*P*_5_ with retention time 21–22 min and *neo*-Ins*P*_6_ with retention time 26.9 min are overlaid

Using the post-column complexation method (Phillippy and Bland 1988) and a CarboPac PA-200 column eluted with a gradient of HCl, we were able to resolve in order of increasing retention time, *neo*-Ins*P*_6_, *muco*-Ins*P*_6_, 1D-*chiro*-Ins*P*_6_, *myo*-Ins*P*_6_ and *scyllo*-Ins*P*_6_ (Fig. 5a), all samples obtained from the laboratory of the late Dennis Cosgrove. We assume that the *muco*-Ins*P*_6_ was that made by chemical phosphorylation of the inositol (Cosgrove 1975). The gradient shows a strongly sloping baseline arising from the acid eluent and this UV absorbance can be negated when using methanesulfonic acid as the eluent (Blaabjerg et al. 2010); however, at least up to 0.6 M, methanesulfonic acid was not a strong enough eluent to elute *scyllo*-Ins*P*_6_ from this column.

### Separation of *neo*-inositol phosphates

Methanesulfonic acid was, however, suitable for separation of *neo*-Ins*P*s: *neo*-Ins*P*_4_s, *neo*-Ins*P*_5_s and *neo*-Ins*P*_6_ (Fig. 5b), again, these compounds were likely produced by chemical phosphorylation of *neo*-inositol with sodium trimetaphosphate (Cosgrove 1969a). Irving (1980) reported that *neo*-Ins*P*_6_ was a good substrate of both the *Pseudomonas* (sp. unknown) bacterium (SB_2_) phytase of Cosgrove (Cosgrove et al. 1970) and *A. ficuum* phytase (Irving and Cosgrove 1974). We note that there are nine possible *neo*-Ins*P*_4_s, comprised of three pairs of enantiomers and three *meso*-compounds and there are three possible *neo*-Ins*P*_5_s (Thomas et al. 2016). Of the *neo*-Ins*P*_5_s, two: 1L-*neo*-Ins(1,2,3,4,5)*P*_5_ = 1D-*neo*-Ins(1,2,3,5,6)*P*_5_ and 1D-*neo*-Ins(1,2,3,4,5)*P*_5_ = 1L-*neo*-Ins(1,2,3,5,6)*P*_5_ are a pair of enantiomers, the third is the *meso*-compound *neo*-Ins(1,2,3,4,6)*P*_5_. In the absence of chiral HPLC methods for separating enantiomers of inositol phosphates, the separation of two peaks of *neo*-Ins*P*_5_ from the Cosgrove samples (Fig. 5b) is all that is achievable, but nevertheless could be diagnostic in studies of *neo*-Ins*P*_6_ transformation in soils.

### Separation of inositol pentakisphosphates; the initial products of phytase action on inositol hexakisphosphate

**Fig. 6:**
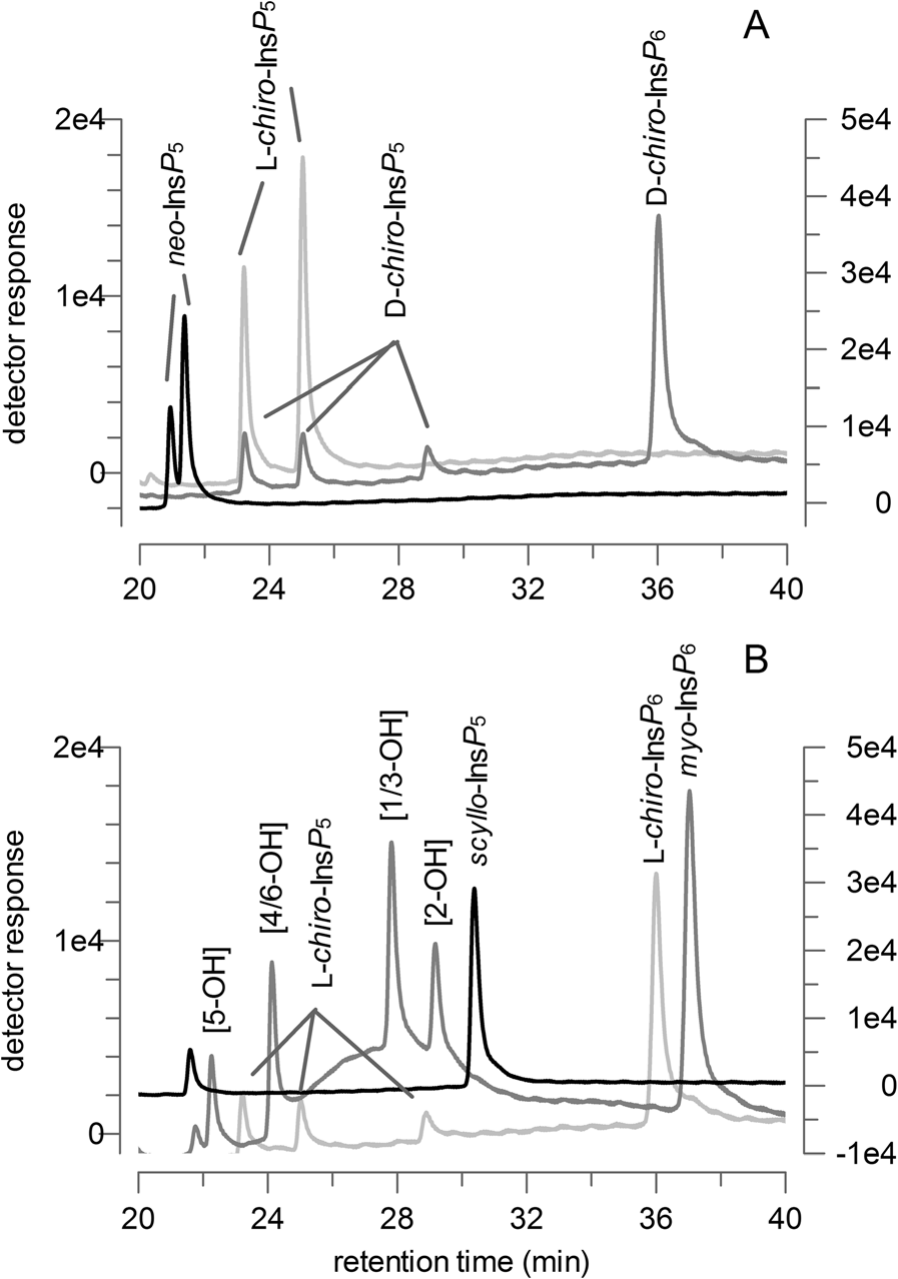
HPLC separation of soil-representative inositol pentakisphosphates. A Dionex CarboPac PA200 column was eluted with a gradient of methanesulfonic acid. Inositol phosphates were obtained from the laboratory of the late DJ Cosgrove. **a** The traces from individual injections of different inositol phosphate samples: a 1D-*chiro*-Ins*P*_6_ sample with three 1D-chiro-Ins*P*_5_s, a 1L-chiro-Ins*P*_5_ sample with two 1L-*chiro*-Ins*P*_5_s and a *neo*-Ins*P*_5_ sample. **b** The traces from individual injections of different inositol phosphate samples: a 1L-*chiro*-Ins*P*_6_ sample with three 1L-*chiro*-Ins*P*_5_s, a *scyllo*-Ins*P*_5_ sample and an acid hydrolysate of *myo*-Ins*P*_6_ with all four resolvable *myo*-Ins*P*_5_. **a**, **b** Traces are offset on the Y-scale (left or right) to aid visualisation

Finally, we show (Fig. 6) the separation of a variety of Ins*P*_5_s including the four separable peaks of *myo*-Ins*P*_5_, identified in Fig. 6b by the position of the single hydroxyl; the two enantiomeric pairs 1D-1/3-OH and 1D-4/6-OH, indicated [1/3-OH] and [4/6-OH] on the figure, cannot be separated into individual enantiomers on non-chiral HPLC. Figure 6b also shows the single *scyllo*-Ins*P*_5_ and several Ins*P*_5_s present in a 1L-*chiro*-Ins*P*_5_ sample. There are three possible 1L-*chiro*-Ins*P*_5_s and, similarly, three possible 1D-*chiro*-Ins*P*_5_s. Minor *chiro*-Ins*P*_5_ peaks with common retention times were observed in the 1D-*chiro*-Ins*P*_6_ sample (Fig. 6a) and the 1L-*chiro*-Ins*P*_5_ sample (Fig. 6b), with two of the three co-eluting precisely with peaks in a 1L-*chiro*-Ins*P*_5_ sample (Fig. 6a). Clearly, this column/eluent combination has great resolving power for all the Ins*P*_5_s expected of soil samples.

## Discussion

Inositol phosphates are major components of total soil phosphate and commonly the dominant organic phosphates in these environments (Turner et al. 2002). Despite their abundance, the origins of *neo*-, 1D-*chiro*- and *scyllo*-inositol hexakisphosphates in soils are poorly defined. It is plausible that they derive from the considerable inputs to soil of plant-derived *myo*-inositol hexakisphosphate, representing up to several percent of seed dry weight (Raboy 2003). It has been estimated that the sequestration of phosphorus in the *myo*-inositol hexakisphosphate, phytate, reserves of cropped organs of plants is equivalent to the per annum application of phosphorus as fertilizer to soils (Lott et al. 2000). It is remarkable therefore that we do not know, how plant-derived phytate is, likely, converted to other forms (epimers) of inositol phosphates, nor whether the processes are organismal or geochemical.

The epimerization of unsubstituted inositols by unsterilized soil is well described (L’Annunziata and Gonzalez 1977) and pathways by which *myo*-inositol and s*cyllo*-inositol are converted have been engineered in *Bacillus subtilis* (Kang et al. 2017; Tanaka et al. 2013; Yamaoka et al. 2011). Similarly, *Geobacillus kaustophilus* HTA426, has three dehydrogenases that are capable of acting as epimerases to interconvert *myo*-, *scyllo*-, and 1D*-chiro*- inositols (Yoshida et al. 2012). The American cockroach*, Periplaneta americana*, was reported to possess epimerase activity producing *neo*-inositol. (Hipps et al. 1973). Plants have the ability to epimerize a number of methylated inositols, reviewed (Thomas et al. 2016). These studies seem to suggest that epimerase activity is restricted to inositols lacking phosphate substituents. It is intriguing therefore that *scyllo*-phosphoinositides were detected in barley (Narasimhan et al. 1997) and that highly phosphorylated inositol phosphates and diphosphoinositol phosphates containing *neo*- rather than *myo*-inositol are the major form of inositol phosphate in *Entamoeba histolytica* (Martin et al. 2000), despite earlier suggestion to the contrary (Martin et al. 1993).

Because the axial 2-phosphate is the last phosphate added in the synthesis of *myo*-inositol hexakisphosphate in plants (Brearley and Hanke 1996) and *Dictyostelium discoideum* (Stephens and Irvine 1990) it is possible to use the enzyme catalyzing this step to make *myo*-inositol 1,[^32^P]2,3,4,5,6-hexakisphosphate. Moreover, because most phytases of plant, bacterial or fungal origin do not remove this phosphate until very late in the sequence of *myo*-inositol hexakisphosphate degradation, if at all (Konietzny and Greiner 2002), the ^32^P label will be retained in successive *myo*-Ins*P*_5_, Ins*P*_4_, Ins*P*_3_ and Ins*P*_2_ products of dephosphorylation. Consequently, addition of ^32^P-labeled *myo*-Ins*P*_6_ to soils will, by simple chromatography using the methods elaborated here, allow researchers to study *myo*-inositol hexakisphosphate turnover in their soil of choice.

From a practical perspective, the use of a high energy β emitter such as ^32^P allows facile detection of radioactivity in column eluates by on-line Cerenkov counting, obviating the requirement for the addition of scintillation fluid. The use of flow-detectors therefore allows for simple collection of radiolabelled fractions, albeit in high salt concentrations required to elute highly polar inositol phosphates. For phosphate eluents, it is a simple exercise to desalt the collected fractions on Dowex AG1 X8 resin with volatile ammonium formate / formic acid mixtures, subsequently removed by freeze-drying (Stephens 1990; Brearley et al. 1997), while for HCl eluents the HCl can be removed directly by freeze-drying.

From a diagnostic perspective, because different phytases produce different *myo*-Ins*P*_5_ products (after all, this is the explanation of their classification e.g. as D3, *Aspergillus*; D6, *E.coli* or D5, lily pollen alkaline phytases (Konietzny and Greiner 2002)), simple analysis of products at the level of *myo*-Ins*P*_5_ will allow identification of the likely class of enzyme predominantly responsible for initial degradation of *myo*-Ins*P*_6_ in different soils. Indeed, it will be fascinating to correlate *myo*-inositol hexakisphosphate degradation products with metagenomic characterization of phytase and micoorganism abundance in different soils of the sort recently described (Neal et al. 2017).

In consideration of the routes of degradation of other inositol hexakisphosphates, the other labeled isomers that we describe will be of particular value. Perhaps unsurprisingly, we note the seminal work of Cosgrove (1969a, 1970) and Irving and Cosgrove (1971) in characterization of products of dephosphorylation of *myo*-inositol-, *scyllo*-inositol-, and 1D-*chiro*-inositol hexakisphosphate by a bacterial phytase, and similar studies of wheat phytase by Lim and Tate (1971, 1973) following the methods of Tomlinson and Ballou (1962). These works, reviewed (Irving 1980), show that the axial phosphates of *myo*- and 1D-*chiro*-inositol hexakisphosphates are the last to be removed by plant, bacterial and fungal phytases where tested. Similar conclusions can be drawn for fungal phytase action on 1L-*chiro*-inositol hexakisphosphate (Adelt et al. 2003). With these observations in mind, the use of the labeled Ins*P*_5_ and Ins*P*_6_ species and the powerful separation approaches identified in the foregoing should allow for facile assessment of the exchange of phosphate between different inositol phosphates in soil contexts.

## Electronic supplementary material

Supplemental Figure 1 The structures of *myo*-, *neo*- *scyllo*- and 1D-*chiro*-inositols. The projections shown are a Mills projection and a three-dimensional structure. Carbon atoms in the ring are numbered according to the IUPAC/IUPAC-IUB recommendations (1973, 1977). Symmetry elements are shown in the Mills projection: planes of symmetry are indicated by dashed lines marked with an asterisk, rotational axes of symmetry are shown by dashed lines marked with symbol, o. The other dashed lines represent apparent symmetry elements that are not real (Thomas et al. 2016). *Myo*-inositol is shown in 1L- notation. (GIF 125 kb)

High resolution image (TIFF 92 kb)

Supplemental Figure 2 The structures of *myo*-, *neo*- and 1D-*chiro*-inositol phosphate substrates and products of AtIP5 2-K. Three-dimensional structures are shown. Carbon atoms in the ring are numbered according to the IUPAC/IUPAC-IUB recommendations (1973, 1977). A,B, for *myo*-Ins(1,3,4,5,6)*P*_5_, 1L- notation is used to number the carbons, but note that the product is a *meso*-compound. B, C, D, for *neo*-Ins(1,3,4,6)*P*_4_, the substrate and products are *meso*-compounds. E, F, G, for D-*chiro*-Ins(2,3,4,5)*P*_4_, the substrates and products are chiral. (GIF 310 kb)

High resolution image (TIFF 2927 kb)

## Abbreviations

HEPES: 4-(2-hydroxyethyl)-1-piperazineethanesulfonic acid
HPLC: High pressure liquid chromatography
i.d.: Internal diameter
