# Supplementary figures and images for: Simple synthesis of 32P-labelled inositol hexakisphosphates for study of phosphate transformations
Source: Plant Soil. 2017 Jun 27;427(1):149–61. doi: 10.1007/s11104-017-3315-9 (PMC5984642; doi:10.1007/s11104-017-3315-9)

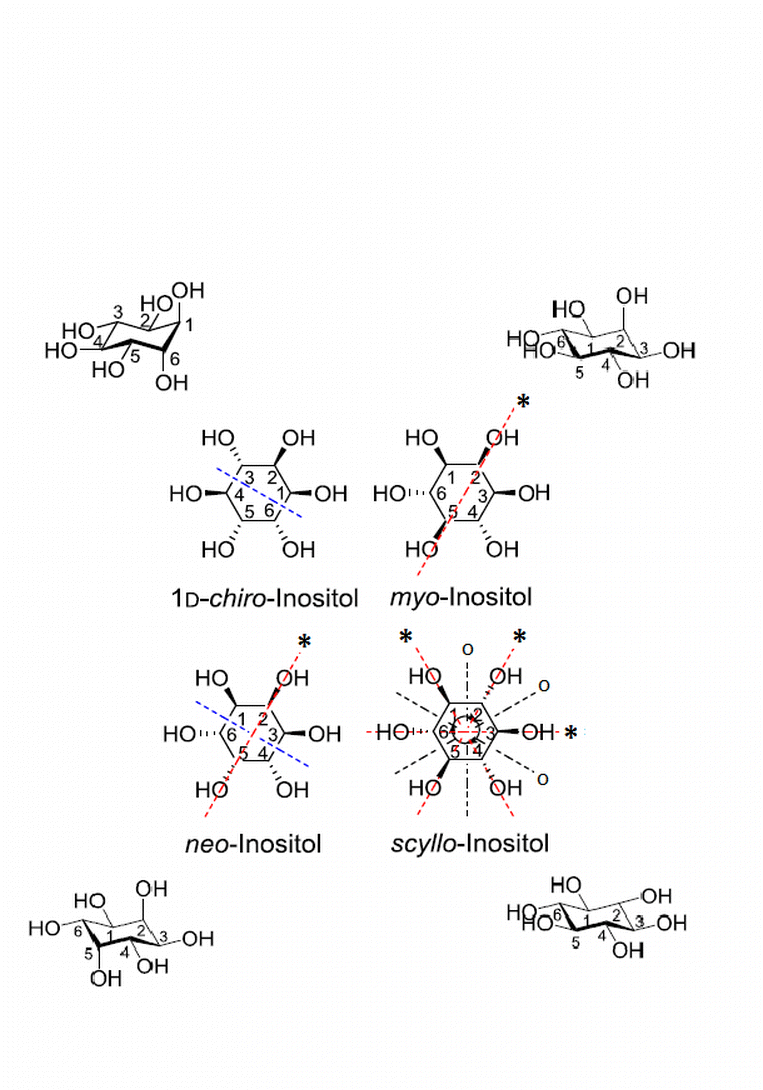

Supplement: Supplementary file 1 — The structures of myo-, neo- scyllo- and 1D-chiro-inositols. The projections shown are a Mills projection and a three-dimensional structure. Carbon atoms in the ring are numbered according to the IUPAC/IUPAC-IUB recommendations (1973, 1977). Symmetry elements are shown in the Mills projection: planes of symmetry are indicated by dashed lines marked with an asterisk, rotational axes of symmetry are shown by dashed lines marked with symbol, o. The other dashed lines represent apparent symmetry elements that are not real (Thomas et al. 2016). Myo-inositol is shown in 1L- notation. (GIF 125 kb) [file 11104_2017_3315_Fig7_ESM.gif]

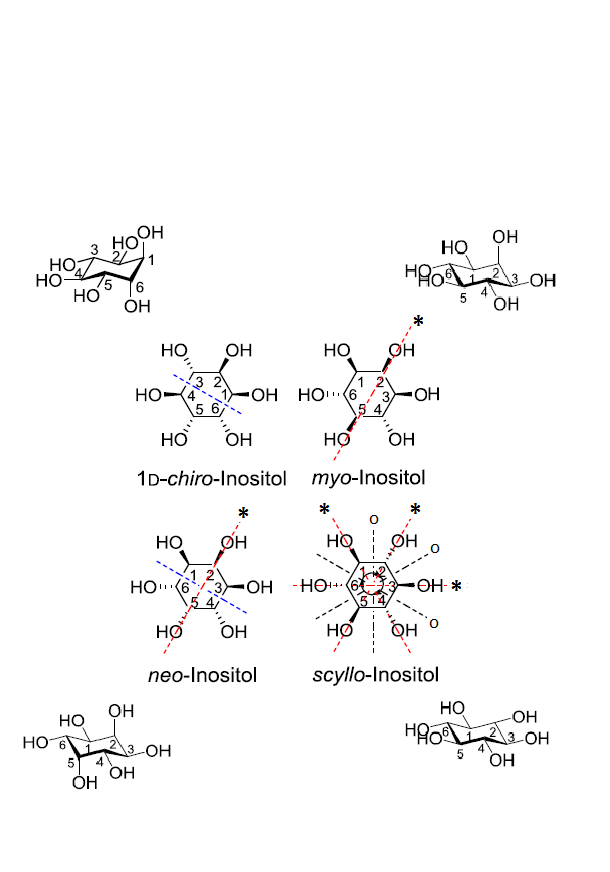

Supplement: Supplementary file 2 — High resolution image (TIFF 92 kb) [file 11104_2017_3315_MOESM1_ESM.tif]

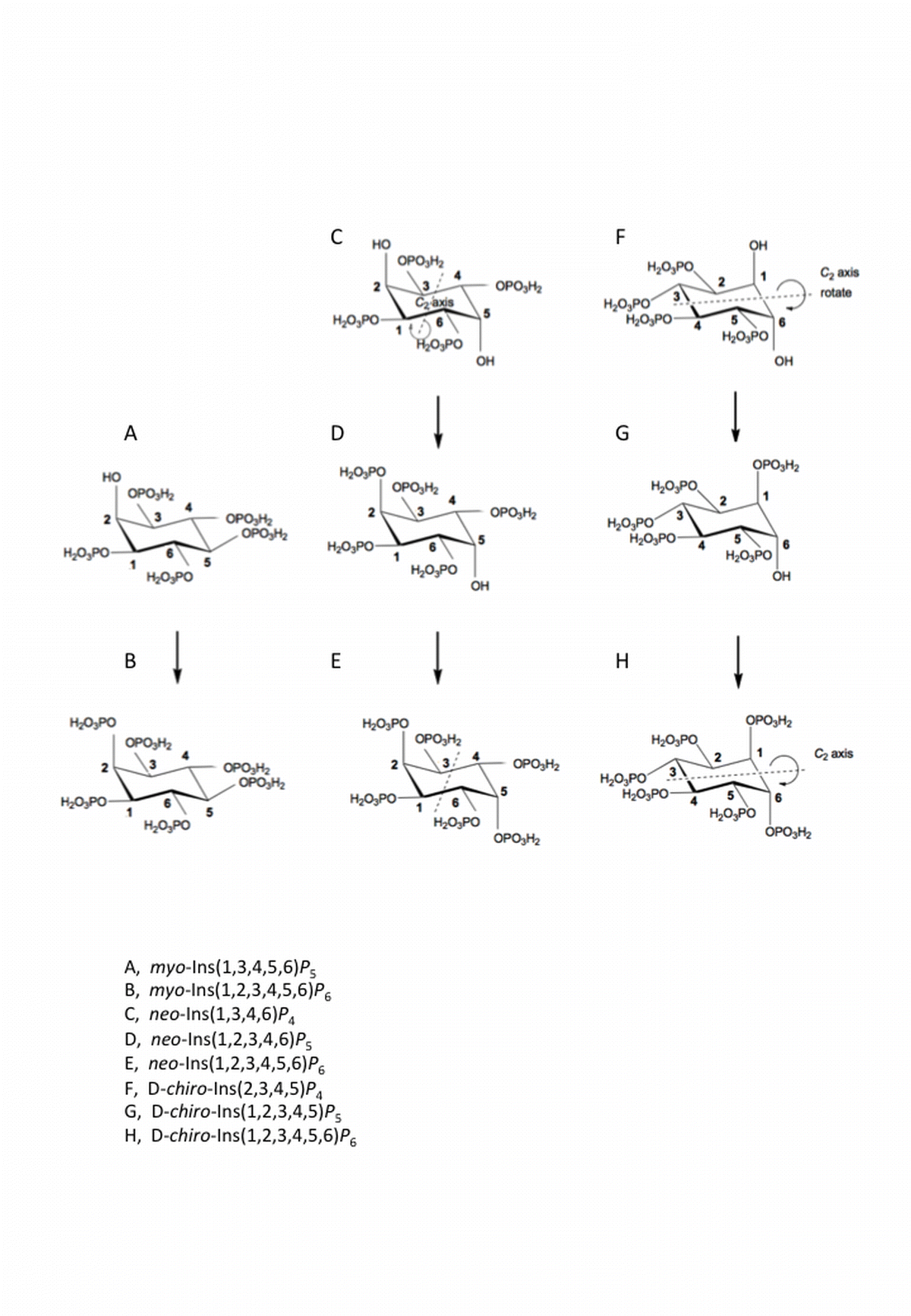

Supplement: Supplementary file 3 — The structures of myo-, neo- and 1D-chiro-inositol phosphate substrates and products of AtIP5 2-K. Three-dimensional structures are shown. Carbon atoms in the ring are numbered according to the IUPAC/IUPAC-IUB recommendations (1973, 1977). A,B, for myo-Ins(1,3,4,5,6)P 5, 1L- notation is used to number the carbons, but note that the product is a meso-compound. B, C, D, for neo-Ins(1,3,4,6)P 4, the substrate and products are meso-compounds. E, F, G, for D-chiro-Ins(2,3,4,5)P 4, the substrates and products are chiral. (GIF 310 kb) [file 11104_2017_3315_Fig8_ESM.gif]

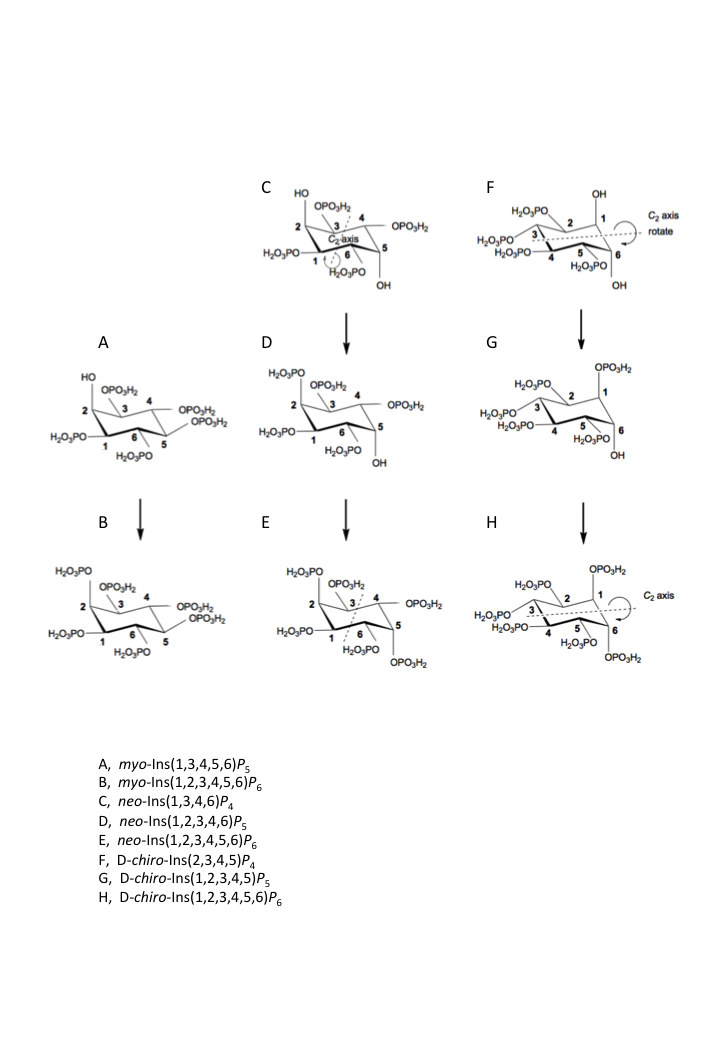

Supplement: Supplementary file 4 — High resolution image (TIFF 2927 kb) [file 11104_2017_3315_MOESM2_ESM.tiff]
